# First results from the study ‘Disease knowledge and information needs - Diabetes mellitus (2017)’

**DOI:** 10.17886/RKI-GBE-2018-064

**Published:** 2018-06-06

**Authors:** Rebecca Paprott, Christin Heidemann, Lena M. Stühmann, Jens Baumert, Yong Du, Sylvia Hansen, Marie-Luise Zeisler, Johannes Lemcke, Silke Beyhl, Ronny Kuhnert, Christian Schmidt, Lars Gabrys, Andrea Teti, Thomas Ziese, Patrick Schmich, Paul Gellert, Daniela Zahn, Christa Scheidt-Nave

**Affiliations:** 1 Robert Koch Institute, Berlin, Department of Epidemiology and Health Monitoring; 2 Charité - Universitätsmedizin Berlin, Institute for Medical Sociology; 3 Federal Centre for Health Education, Cologne Office for National Education and Communication on Diabetes Mellitus; 4 University of Applied Sciences for Sport and Management, Potsdam; 5 University of Vechta Institute for Gerontology

**Keywords:** DIABETES MELLITUS, TELEPHONE SURVEY, DISEASE KNOWLEDGE, DISEASE PERCEPTION, INFORMATION-SEEKING BEHAVIOUR

## Abstract

Very little research has been undertaken into what people in Germany know about diabetes, the information they may require about the condition, where they look for such information and how they rate the information currently available. In 2017, the Robert Koch Institute (RKI) carried out a nationwide telephone survey aimed at answering these questions. The study entitled ‘Disease knowledge and information needs - Diabetes mellitus (2017)’ focused on people aged at least 18 years. A total of 2,327 people without diabetes and 1,479 people with diagnosed diabetes were interviewed for the study. First results show that 56.7% of people without diabetes and 92.8% of those with diabetes rate their knowledge about the condition as ‘very good’ or ‘good’. People without diabetes were found to have the strongest need for information in terms of ‘lifestyle changes, health promotion and disease prevention’, whereas respondents with diabetes stressed the strongest need for information about ‘treatment and therapy’. Almost a third of respondents without diabetes have actively sought information about diabetes at least once, mostly via print media. Patients with diabetes stated that their general practitioner was their most frequent source of information about the condition. In both groups, about half of respondents reported that they found it difficult to judge the trustworthiness of the information published in the media about diabetes. The results of the study form part of the German National Diabetes Surveillance, which is coordinated by the RKI. The data are also intended to be used by the Federal Centre for Health Education to develop a strategy to improve the information provided about diabetes.

## 1. Introduction

Diabetes mellitus is a chronic metabolic disorder characterised by elevated blood-sugar levels [1]. The condition can be divided into a number of different types. Type 1 diabetes is an autoimmune disease that is thought to be primarily caused by a genetic predisposition and environmental factors [2, 3]. In contrast, genetic but also individual, social and contextual factors such as the person’s living environment or health care conditions as well as the way in which these factors interact with one another play a role in the development of a much more common form: type 2 diabetes [4-6]. A family history of diabetes, lifestyle and individual patterns of behaviour such as smoking, a lack of physical activity and dietary aspects are associated with a higher risk of type 2 diabetes [4-6].

**Info box 1: Study ‘Disease knowledge and information needs - Diabetes mellitus (2017)’**

**Data holder:** Robert Koch Institute

**Objectives:** To provide reliable information from adults in Germany about their perception of the risk associated with diabetes mellitus, their perception and knowledge of the condition, their information needs and information-seeking behaviour, the subjective burden of diabetes and the quality of care

**Survey method:** Telephone interview

**Population:** The German-speaking general population aged at least 18 years with or without a diagnosis of diabetes living in private households who were reachable by telephone

**Sampling:** Individual private households in Germany that are reachable using randomly selected landline or mobile telephone numbers

**Sample size:** 2,327 people without diabetes (1,313 women, 1,014 men) and 1,479 people with diagnosed diabetes (740 women, 739 men)

**Response rate:** 17.9% (of all households reachable by telephone in Germany)

**Survey period:** September - November 2017

**Data protection:** the participants were informed about the study’s aims and content and provided their informed consent to participate in the study

More information in German is available at:

www.rki.de/krankheitswissen_diabetes

Individual patterns of behaviour can be influenced by a variety of subjective factors which may play a key role in the condition but have often been under-studied. These factors include an individual’s subjective perception of their risk of developing diabetes [7], their knowledge about the disease [8], their perception of the condition [9] and their health-related information behaviour [10, 11]. In the context of this study, health-related information behaviour refers to the issues of whether people actively seek information about health related topics and, if so, the sources of information that they turn to. Ultimately, contextual factors such as social support and the conditions associated with care also affect people’s risk of developing diabetes [12]. Moreover, international studies have shown that the perception of the risk associated with complications linked to diabetes, the way in which people deal with the condition, the social support they receive, as well as various aspects linked to care and access to the information required to deal with the disease can influence the way in which the disease develops over time [13-19].

According to data from the Robert Koch Institute (RKI), an estimated 6.7 million adults in Germany are affected by diagnosed or undiagnosed diabetes [20, 21]. Although diabetes can be treated with medication, the disease is still associated with serious complications and increased risk of mortality [22, 23]. In addition, diabetes also incurs a large amount of direct and indirect costs to society, mainly because of these complications [24]. In order to tailor healthcare services towards people’s needs and requirements, it is essential that data be collected on the burden caused by the disease, on people’s knowledge about the condition, and the need for information and support among people with diabetes. Furthermore, data from the general population - in particular on knowledge about the disease, disease perception and, above all, information needs and information-seeking behaviour - are vital if measures in population-wide health promotion and the primary prevention of type 2 diabetes are to be effective.

At the present time, only a limited number of population-representative data are available on these issues at the national level for people with or without diagnosed diabetes [25-29]. Moreover, the data on information-seeking behaviour and information needs [26, 28] is fragmentary and only a small proportion of participants with diabetes took part in these studies [26, 29]. The ‘Disease knowledge and information needs - Diabetes mellitus (2017)’ study (Info box 1), therefore, aimed to close these gaps in the data. In order to do so, the study collected data on knowledge about the disease, disease perception, the risk of diabetes and the risk associated with diabetes-related complications, subjective aspects of the disease as well as specific diabetes-related information (Info box 2). This article aims to set out the study’s first descriptive findings for the key topics of knowledge about disease, disease perception and diabetes-related information from people with and without diabetes.

Finally, the study also provides a foundation with which to develop the educational and communications-related strategy on diabetes that is currently being drawn up by the Diabetes Office of the Federal Centre for Health Education (BZgA). This strategy is to reflect people’s current needs and will be tailored to specific target groups [30]. The goal is to improve diabetes prevention, including primary prevention (reducing the incidence of diabetes), secondary prevention (early detection of previously unknown diabetes) and also tertiary prevention (prevention of the progression of diabetes). Furthermore, the results of the study have been integrated into the National Diabetes Surveillance which is currently being established at the RKI [31].

**Info box 2: Diabetes-related information**

Diabetes-related information includes the following aspects:

1. **Perceived level of information**
  How informed do respondents feel about different diabetes-related topics?
2. **Subjective information needs**
  Which diabetes-specific topics do respondents require more information about?
3. **Information-seeking behaviour**
  Are respondents actively looking for information and, if so, which sources do they turn to?
4. **The perceived trustworthiness and clarity of information about diabetes** published in the media

## 2. Methods

### 2.1 Study design and sample

**Figure 1:**
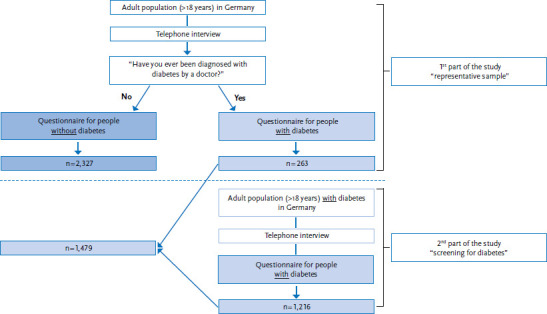
Flowchart for sampling in the study ‘Disease knowledge and information needs - Diabetes mellitus (2017)’ Own diagram

The study ‘Disease knowledge and information needs - Diabetes mellitus (2017)’ (Info box 1) was undertaken in two parts that were conducted using a standardised telephone survey (Figure 1). The first part of the study focused on the German-speaking general population aged at least 18 years (including people with diabetes); the second part only included people with diabetes. Sampling was undertaken using the ADM Telephone Survey System, which is provided by Arbeitskreis Deutscher Markt- und Sozialforschungsinstitute e.V. (ADM) [32]. This system can be used together with a special procedure to generate all usable German landline and mobile phone numbers. This even includes numbers that are not registered in public telephone directories. The study applied the dual-frame principle, which provided a sample of 60% landline numbers and 40% mobile telephone numbers. This method ensured a representative sample of all potentially reachable private households in Germany. A detailed description of the methodology used by the RKI for telephone interviews and the sampling applied in this case will be provided in an article that is due to be published in 2018 in the Journal of Health Monitoring [33].

Interviewees (target persons) for the first part of the study were randomly selected from multi-person households using the Kish Selection Grid method. For this procedure, all potential target persons have the same probability of selection. The number of adults in the household in question and their age are first determined in order to draw up a particular sequence of these individuals. An algorithm is then employed as part of computer-assisted random selection to select the target person for interview. In the case of this study, the aim was to gain a sample of 2,500 people without diabetes with which to assess the different groups within the general population and to provide stratified analysis with the lowest possible level of error tolerance.

The second part of the study focused on people with a physician diagnosed diabetes. The aim was to gain a sample of 1,500 individuals (including those with diabetes from the first interview part) also in order to ensure that stratified analysis had the lowest possible tolerance of error. The study was implemented using a direct screening procedure to find participants for the category ‘people with diabetes’. In these cases, the person who answered the call (the contact person) was asked whether they or another person in the household had ever been diagnosed with diabetes by a physician (the target person). If a household had more than one potential target person, in other words, more than one person with a physician diagnosed diabetes, the target person was randomly selected using the Kish selection grid [33].

### 2.2 Study procedure

Two questionnaires were developed for the study: one for people with a physician diagnosed diabetes, and one for those without. The two questionnaires deliberately used overlapping questions so that the results for people with diabetes could be compared to those without the condition. Wherever possible, the questions were selected using validated German-language instruments. In case only English-language instruments were available they were translated into German before translating them back into English (forward-backward translation) [34, 35]. The translations were shown to be very similar to the original texts.

A number of further questions had to be newly developed for the study to cover other topics of interest. A detailed description of this process and the psychometric properties of the selected constructs and instruments is currently being summarised for a methodological publication. Cognitive testing was conducted to assess how understandable the questions and certain terminology were. Furthermore, in order to assess the completeness of the questionnaires, find out how long the interviews would take, and address any remaining ambiguities, a standard pretest was carried out in the field between the 23 August and 17 September 2017 (203 participants: 28 with diabetes, 175 without). The results from the pretest were used to shorten the questionnaires to an average of 32 minutes (for respondents without diabetes) and 43 minutes (for respondents with diabetes) and to adapt the questions.

The first part of the main study was conducted between 18 September and 31 October 2017, with the second taking place between 1 November and 30 November 2017. 2,592 interviews were conducted for the first part of the study (the representative part). This figure also includes 263 people who had been diagnosed with diabetes. The response rate, which was calculated using criteria from the American Association for Public Opinion Research (AAPOR) (in this case: response rate 3, proportion of interviews conducted in relation to all probable households in the population) was 17.9% [36]. 1,216 interviews were conducted for the second part of the study.

Two respondents were excluded from the first part of the study due to an unclear answer to the question ‘Have you ever been diagnosed with diabetes by a doctor’ (‘don’t know’) or missing information about the federal state of residence (this was needed to calculate the weighting factors). The final sample, therefore, consisted of 2,327 people without a diagnosis of diabetes and 1,479 people with a physician diagnosed diabetes.

### 2.3 Definition of diabetes

Participants with diabetes were identified using the question ‘Have you ever been diagnosed with diabetes by a doctor?’. This group was further subdivided into: 1) Diabetes in the last 12 months: in this case, a participant either answered ‘Yes’ to the follow-up question as to whether they had had diabetes in the last 12 months, or stated that they were undergoing medical treatment for diabetes (n=1,396); 2) No diabetes in the past 12 months: the respondent answered ‘No’ to the question about whether their diabetes had been present in the last 12 months, and also provided no indication that they were currently undergoing medical treatment for diabetes (n=89); 3) Gestational diabetes: the respondent answered ‘Yes’ to the question as to whether they were pregnant at the time of their diagnosis (n=52). A current case of gestational diabetes was identified by asking whether diabetes had been diagnosed during a current pregnancy (n=1). Of the female participants who had ever been diagnosed with gestational diabetes, twelve women had type 2 diabetes at the time of the study.

### 2.4 Concepts and instruments

The study collected information on disease knowledge, disease perception, diabetes risk, the risk of complications, diabetes-related information, health-related behaviour and lifestyle, health care services utilisation, the characteristics of disease, diabetes self-management, the subjective burden of disease and mental health. An overview of all of the subject areas and instruments employed in the survey can be found in Annex 1. This article presents the first results for selected topics, including disease knowledge, disease perception and diabetes-related information.

#### Survey instruments used for respondents without diabetes

In order to study knowledge about disease, data was collected about the respondents’ perceived level of knowledge about diabetes [37] and their objective level of knowledge about the disease. Annex 2 provides a list of the questions that were posed and acceptable response options.

The topic of disease perception included an assessment of whether the respondents’ considered diabetes to be a serious disease [38], and whether they believed that diabetes was a condition that people had for the rest of their life [39]. In addition, data was also collected on various aspects of stigmatisation [26, 40].

In the case of diabetes-related information, data was gathered on the respondents’ perceived level of information and personal need for information in terms of the causes of diabetes, the course of the disease, its treatment and therapy, the complications it can lead to, lifestyle changes as well as health promotion and prevention [41]. With regard to information-seeking behaviour, participants who had previously informed themselves about diabetes were asked which sources of information they had used to do so [29]. The remaining participants were asked where they gained information about general health issues [29]. In each case, several sources of information could be provided.

Respondents who had already actively sought information about diabetes were also asked about their view of the trustworthiness and clarity of the information about diabetes that is published in the media [42].

#### Survey instruments used for people with diabetes

Respondents with diabetes were asked the same questions about their level of knowledge about diabetes and about the severity of diabetes that were posed to respondents without the condition. However, the question as to whether a respondent believed that they would have diabetes for the rest of their life [43] and the questions linked to stigmatisation [26, 40] were only posed to respondents who had stated that they had had diabetes in the last 12 months. These questions only differed in perspective from those posed to people without diabetes: people with diabetes were not only asked about diabetes in general, but also about their own condition. Moreover, the questions on their perceived level of information, individual information needs and sources of information were posed for a broader range of topics than for people without diabetes. For example, data was also collected on support services, helplines and sources of information [41]. Finally, both groups were also asked the same questions about the trustworthiness and clarity of the information published in the media.

### 2.5 Statistical analysis

In order to ensure that a sample is representative of the general population, it is important to take into account all relevant population groups, especially those who are less willing to participate in the study. As such, this study also employed a weighting factor. First, the distribution of household sizes in the sample was adjusted to reflect the known distribution within the population in Ger many (as of 31 December 2016) [44]. The sample was then adjusted to take into account the different probabilities of selection. This was followed by a standard method of calculation that is recommended by the ADM and that takes into account the study’s dual-frame design [45]. Finally, adjustment weighting was applied. For the representative part of the study (the first section with 2,591 interviews including the 263 interviews of people with diabetes), a gradual weighting in terms of age × gender (in 7 × 2 steps), education x gender (in 3 × 2 steps) and federal state (in 16 steps) was implemented to ensure that these characteristics were distributed in a manner that was comparable to the German-speaking, resident population aged at least 18 years. Data supplied by the Federal Statistical Office were used (as of 31 December 2016) to make these adjustments [44].

As the Federal Statistical Office’s data do not enable conclusions to be drawn about individuals with diabetes among the German-speaking resident population aged at least 18 years they could not be applied to the sample of respondents with diabetes (1,479 interviews including 1,216 from the screening conducted for diabetes and 263 interviews from the representative part of the survey). Instead, a gradual weighting was implemented for age x gender (in 6 × 2 levels) and education × gender × age (in 3 × 2 × 2 levels) to reflect the distribution of people with diabetes identified by the German Health Update study (GEDA) 2012 (n=1,828) [46]. The GEDA 2012 sample was used because the study applied a comparable methodology. The distributions identified by GEDA 2012 were assumed to correspond to those within the current German-speaking resident population aged at least 18 years.

Finally, a third weighting factor was employed to enable comparisons to be made between respondents with and without diabetes in the overall data set. This was necessary because the screening method used in the study led to a significant overrepresentation of respondents with diabetes in the overall sample. The weighting factor adjusted the ratio of respondents with and without diabetes to ensure that it reflected the level identified by the representative part of the study; as such, the proportion of respondents with diabetes in the overall data set was the same as the proportion identified in the representative part of the study (9.7%).

All results presented here were calculated using the respective weighting factor. Averages or percentages (including 95% confidence intervals, 95% CI) were calculated using SAS (Version 9.4, SAS Institute Inc., Cary, NC). The results from both samples are presented as totals and separately for women and men (Annex 3). In addition, differences in terms of educational group (low, middle and high) were examined (Annex 4). These groups were classified using the CASMIN scale (Comparative Analysis of Social Mobility in Industrial Nations) [47], which takes both general and vocational training into account.

## 3. Results

### 3.1 Population without diabetes

In total, 2,327 people (1,313 women, 1,014 men) between the age of 18 and 97 years without a previous diagnosis of diabetes participated in the telephone survey. Their average age was 50.4 years (women 51.7 years, men 48.3 years).

#### Knowledge about the disease

**Figure 2:**
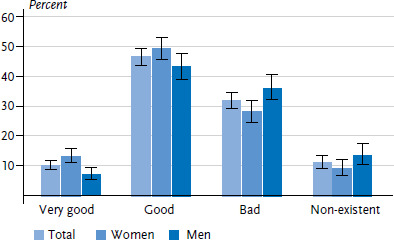
Self-assessed level of knowledge about diabetes among adults without diagnosed diabetes according to gender (n=1,305 women, n=1,008 men) Source: Disease knowledge and information needs - Diabetes mellitus (2017)

More than half of the participants without diabetes perceived their knowledge about the disease as very good or good (Figure 2). The proportion of people who perceived their own level of knowledge as very good was almost twice as high among women as men. Respondents with low levels of education were more likely to rate their knowledge of diabetes as ‘non-existent’ than people with middle or high levels of education. Furthermore, respondents with low or middle levels of education also perceived their knowledge of diabetes as very good less frequently than those with high levels of education (Annex 4 Table 3).

A total of six questions were used to collect data about the respondents’ objective level of knowledge about diabetes (Annex 3 Table 1). The participants were most frequently aware of the fact that people with diabetes had elevated blood-sugar levels (66.2%) and that the symptoms of diabetes usually develop gradually (62.6%). Almost half of the respondents (49.6%) were aware that more people are affected by type 2 diabetes in Germany than type 1. By contrast, less than a third of respondents (31.3%) knew that diabetes was not a blood cell disorder. Respondents were the least familiar with type 1 diabetes. Only 26.9% of respondents knew that type 1 diabetes does not disappear with puberty and only 17.2% knew that it cannot be treated with tablets. An educational gradient was identified among answers to the question as to whether diabetes was a blood cell disorder. Four further questions were answered correctly more frequently by respondents with high levels of education than those with low levels of education (Annex 4 Table 1).

#### Disease perception

Almost two-thirds of respondents (65.0%) considered diabetes to be a serious or very serious disease (Annex 3 Table 2). In contrast, only 1.2% of respondents felt that diabetes was not a serious condition, while 12.6% of respondents expressed no opinion on this issue. The results were similar among women and men. Respondents with low levels of education rated diabetes less often as not or moderately serious compared to the two higher educational groups (Annex 3 Table 3).

60.1% of respondents believe that diabetes is a condition that people have for the rest of their lives. However, 28.2% rejected outright or partially disagreed with this statement (Annex 3 Table 2). The results were similar for women and men and across the three educational groups (Annex 4 Table 2).

A small proportion of respondents believed that people with diabetes are discriminated against (Annex 3 Table 2). Overall, 20.6% felt that people with diabetes are often unable to meet their everyday needs, and 28.8% believe that individuals with diabetes cause the disease themselves through an unhealthy lifestyle. Only 14.0% of respondents felt that people with diabetes face disadvantages. No differences according to gender where identified in attitudes towards people with diabetes. Participants with middle or high levels of education were more likely than those with low levels of education to believe that people with diabetes can meet their everyday needs (Annex 4 Table 2).

#### Diabetes-related information

##### Perceived level of information

44.0% of participants felt that they were well or very well informed about the ‘causes of diabetes’; between 36.0% and 41.2% felt the same way about the ‘course of the disease’, ‘treatment and therapy’ and ‘complications’. Furthermore, this was the case with 48.9% of respondents when it came to ‘lifestyle changes, health promotion and disease prevention’. Women considered themselves better informed than men on all issues. Participants with lower and middle levels of education more frequently felt completely uninformed about these issues than those with a high level of education (Annex 4 Table 3).

##### Subjective information needs

**Figure 3:**
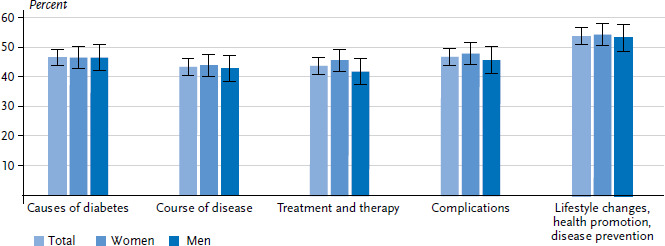
Subjective information needs about diabetes-specific issues among adults without diagnosed diabetes according to gender (n=1,313 women, n=1,014 men) Source: Disease knowledge and information needs - Diabetes mellitus (2017)

The greatest need for information was identified for the issues of ‘lifestyle changes, health promotion and disease prevention’ (Figure 3). No differences were found between women and men in this regard. The need for information on ‘causes of diabetes’, ‘course of the disease’ and ‘complications’, was higher among people with a middle level of education than among those with a high level of education (Annex 4 Table 4). Participants who felt uninformed about the ‘causes of diabetes’, ‘course of the disease’, and ‘complications’ also expressed a greater need for information than those who saw themselves as well or very well informed (data not shown).

##### Information-seeking behaviour

Almost a third (31.0%) of respondents stated that they had actively sought information about diabetes at least once. However, this was more common among women (37.0%) than men (24.7%). Printed information such as brochures, newspapers, magazines and books were the most commonly used source of information (83.9%), followed by websites on health and diabetes (49.3%), general practitioners and other doctors (47.2%) as well as radio or television broadcasts (43.4%). In this context, women more frequently cited printed information than men (88.9% compared to 75.9%). Respondents with low levels of education more frequently sought information from their general practitioner or other doctors than respondents with middle or high levels of education (65.9%, 43.4% and 41.8% respectively; data not shown). The most frequently mentioned sources of information on health issues in general were general practitioners or other doctors (72.0%), followed by printed information (69.6%), radio or television broadcasts (60.0%) and websites covering health issues (53.7%). Participants with middle or high levels of education use websites more frequently as sources of information than those with low levels of education (70.6%, 60.0% and 35.5% respectively; data not shown).

##### The perceived trustworthiness and clarity of information

**Figure 4:**
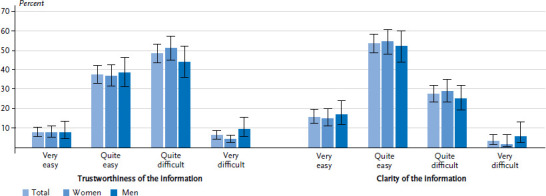
**Perceived level of trustworthiness and clarity of information among adults without diagnosed diabetes according to gender.** Only participants who had already actively sought information about diabetes were asked this question **(n=550 women, n=318 men)** Source: Disease knowledge and information needs - Diabetes mellitus (2017)

More than half (54.7%) of people who had previously actively sought information about diabetes found it difficult or very difficult to judge whether the information published in the media about the condition was trustworthy (Figure 4). In contrast, 69.3% of respondents found it very easy or fairly easy to understand this information. No gender or educational differences were identified (Annex 4 Table 7).

### 3.2 Population with diabetes

In total, 1,479 people (740 women, 739 men) aged between 18 and 96 years with a physician diagnosed diabetes participated in the telephone survey; 263 of these respondents had taken part in the representative part of the study. Data for the prevalence of diabetes in the representative survey was gathered by asking respondents whether they had ever been diagnosed with diabetes; the prevalence rate was 9.7% (8.2%-11.5%). The average age of all participants with diabetes was 64.9 years (women 65.3 years, men 64.5 years). The mean duration of diabetes was 14.3 years (women 13.7 years, men 15.0 years). In total, 1,386 participants (92.1%) who had ever been diagnosed with diabetes had had diabetes during the last 12 months. The prevalence of diabetes in the last 12 months was 8.7% (7.2%-10.5%). Respondents who had diabetes within the last 12 months were also asked about the type of diabetes they were affected by: 14.1% of respondents who had had diabetes over the last 12 months stated that they had type 1 diabetes; 80% that they had type 2 diabetes; 1.4% another type; and 4.6% did not know which type of diabetes they were affected by. In terms of treatment, 90.2% of these respondents stated that they were receiving medication such as tablets, insulin or other injectable hypoglycaemic drugs. 7.4% were only in receipt of treatment through diet and sport, and 2.4% of these respondents said they were not receiving any treatment at all.

####  

##### Knowledge about diabetes

**Figure 5:**
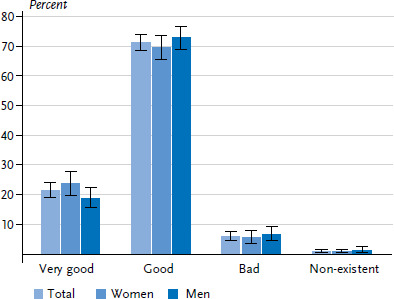
Self-assessed level of knowledge about diabetes among adults with diagnosed diabetes according to gender (n=732 women, n=732 men) Source: Disease knowledge and information needs - Diabetes mellitus (2017)

92.8% of respondents who had received a diagnosis of diabetes at some point viewed their knowledge of the condition as good or very good (Figure 5). The self-assessed level of knowledge was similar between women and men and across the various educational groups (data not shown).

#### Disease perception

Of the respondents who had ever been diagnosed with diabetes, 55.7% viewed the condition as serious or very serious (Annex 3 Table 4). 91.1% of respondents who had diabetes in the last 12 months agreed or completely agreed that they would have diabetes for the rest of their lives (Annex 3 Table 4). The levels of disease perception were similar among women and men and across educational groups (Annex 4 Table 5).

Regarding stigmatisation, 6.1% of respondents who had diabetes over the last 12 months felt that they often faced disadvantages because of their diabetes (Annex 3 Table 4). 22.3% of respondents felt that other people believed that they caused their diabetes themselves due to their unhealthy lifestyles. Men were more likely to agree with this statement than women. Furthermore, respondents with middle levels of education were more likely than those with a high level of education to believe that other people assumed that they would not be able to meet their day-to-day needs (Annex 4 Table 5).

#### Diabetes-related information

##### Perceived level of information

Respondents who had received a diagnosis of diabetes at some point in time felt the least informed about ‘support services, helplines and sources of information’ (Annex 3 Table 5). Only 57.1% of these individuals believed that they were well or very well informed about these issues, although more than three quarters felt that they were well or very well informed about other diabetes-related topics. 72.2% stated that they were well or very well informed about ‘lifestyle changes, health promotion and disease prevention’. No serious differences were identified between women and men. Participants with low levels of education more frequently felt completely uninformed about all of these issues than those with higher levels of education (Annex 4 Table 6).

##### Subjective information

**Figure 6:**
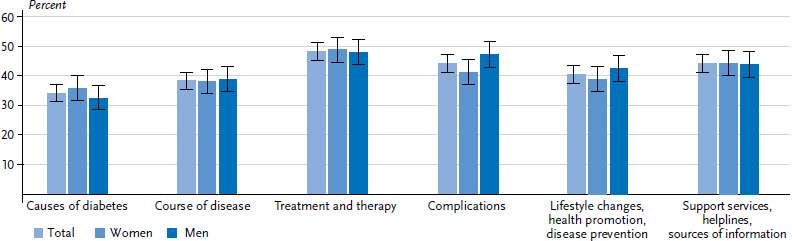
Subjective information needs about diabetes-specific issues among adults with diagnosed diabetes according to gender (n=740 women, n=739 men) Source: Disease knowledge and information needs - Diabetes mellitus (2017)

The area where the need for information was highest was ‘treatment and therapy’; it was lowest for ‘the causes of diabetes’ (Figure 6). Women and men have a similar need for information. Respondents with middle levels of education had a higher need for information about ‘the causes of diabetes’, ‘course of the disease’, ‘treatment and therapy’ and ‘complications’ than respondents with a high level of education. People with a lower level of education had the highest need for information about ‘the causes of diabetes’ (Annex 4 Table 7). On all issues except for ‘lifestyle changes, health promotion and disease prevention’, participants who considered themselves poorly informed or completely uninformed about these issues had a greater need for information than those who considered themselves well or very well informed (data not shown).

##### Information-seeking behaviour

Participants who have been diagnosed with diabetes stated their general practitioners or other doctors who were treating their diabetes as their most frequent source of information (91.8%). This was followed by printed information (83.7%), their diabetologist (59.6%), information programmes for people with diabetes (57.6%) and diabetes advisors (45.6%). Respondents with a low level of education indicated that diabetologists were their most frequent sources of information less frequently than people with high levels of education (54.0% and 67.5% respectively). Similarly, they were less likely than those with a high level of education to emphasise printed information as their main source of information (79.7% compared to 91.0%) or diabetes websites (24.0% compared to 55.3%) (data not shown).

##### Perceived trustworthiness and clarity of information

**Figure 7:**
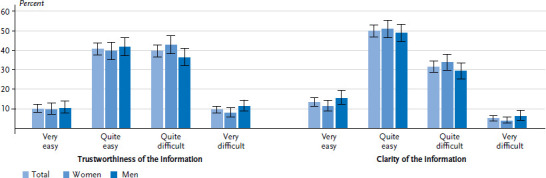
Perceived level of trustworthiness and clarity of information among adults with diagnosed diabetes according to gender (n=740 women, n=739 men) Source: Disease knowledge and information needs - Diabetes mellitus (2017)

About half of the respondents who had ever been diagnosed with diabetes found it difficult or very difficult to judge the trustworthiness of information published in the media about diabetes (Figure 7). Nevertheless, 63.2% stated that they found this information very easy or fairly easy to understand. No differences were identified between women and men on this issue. Respondents in this group with a high level of education stated more frequently that it was easy to understand the information published about diabetes in the media than those with low or middle levels of education (Annex 4 Table 7).

### 3.3 Comparison of the population with and without diabetes

Respondents who had ever been diagnosed with diabetes rated their knowledge of the condition as very good or good more frequently than those without diabetes. In addition, people who had ever been diagnosed with diabetes were less likely to view the disease as very serious or serious than respondents without diabetes. Participants who had ever received a diagnosis of diabetes felt better informed about all topics than those without diabetes. Respondents with diabetes expressed less of a need for information about ‘the causes of diabetes’ and on ‘lifestyle changes, health promotion and disease prevention’; no differences were identified between the information needs of the two groups in terms of the remaining topics. Around half of both the respondents with and without diabetes found it difficult to judge the trustworthiness and clarity of information that is published in the media about diabetes.

## 4. Discussion

The method established at the RKI of using nationwide telephone health surveys was employed to collect comprehensive information from adults in Germany about their knowledge, disease perception, information needs and information-seeking behaviour within the context of diabetes. The study included interviews from adults with a diagnosis of diabetes and those without the condition. In addition to collecting the same information from both samples, specific data was also collected for people with and without diabetes. The results can be stratified according to socio-demographic, psychosocial and - in the case of respondents with diabetes - disease-specific characteristics. The first descriptive results show that the study can help close important information gaps and help provide the foundation for the target-group-sensitive information and communications strategy that is being developed at the BZgA.

### 4.1 Discussion of the first results

It seems that hardly any studies have collected data from the general population about diabetes knowledge or disease perception. The results presented here show that only about half of the surveyed adults without diabetes felt that they were well or very well informed about diabetes. This corresponds to the results of a survey conducted of the general population by forsa, an opinion research institute, on behalf of Krankenkasse Knappschaft in 2016 [28]. The answers to questions about respondents’ knowledge of diabetes also led to the identification of significant gaps in people’s knowledge, some of which are more prevalent among men than women or are associated with a respondent’s level of education. A 2016 study commissioned by the magazine Diabetes Ratgeber, which was carried out by GfK, a society for consumer research, also identified gaps in knowledge among adults without diabetes, such as with regard to a cure and the treatment of the condition [26]. Furthermore, 54% of the present study’s participants stated that they knew next to nothing about the triggers of diabetes [26]; in the forsa survey, 36% of respondents did not know the causes of type 2 diabetes [28]. In this context, the present study shows that only 45% of respondents felt very well or well informed about the causes of diabetes.

In the survey conducted for the Diabetes Ratgeber, 47% of participants without diabetes and 54% of those with diabetes rated the disease as serious. This result differed from our study, where 65% of respondents without diabetes considered the condition to be a serious or very serious condition. This difference is probably explained by the difference in the questions posed by both studies. Our study asked the participants directly whether they considered diabetes to be a serious disease, the Diabetes Ratgeber survey asked participants for their opinion of the following statement: ‘Since diabetes can now be treated well with insulin or tablets, it is no longer a serious illness.’ Our study also found less agreement among respondents with and without diabetes that people with diabetes had caused their condition through their unhealthy lifestyle than was identified by the Diabetes Ratgeber study. Finally, our study also identified a similar proportion of people with diabetes who felt disadvantaged or discriminated against compared to the results of the German ‘Diabetes Attitudes Wishes and Needs 2’ study (DAWN-2, 2012) and the Diabetes Ratgeber study [26, 48].

Very few studies have been conducted into the specific information needs concerning diabetes among people with and without the condition [49]. A recent systematic review by Kuske et al. [50] on information-seeking behaviour shows that people with diabetes most frequently look for information about the treatment process; this is consistent with the results of our study. In terms of socio-demographic differences between information needs, the first results from our study indicate a need for further research. However, this study can make an important contribution to this situation as it provides for in-depth analyses. A systematic review by Pieper et al. from 2015 [27] identified important information needs among the general population in Germany in terms of the causes, prevention, treatment and course of diabetes. In our study, the need for information among people without diabetes was highest when it came to disease prevention. Furthermore, a greater need for information about the causes of diabetes, its course and complications can be found among people who feel less well or not so well informed about these topics. The Diabetes Ratgeber study posed no detailed questions about the need for information; however, about 87% of the study’s respondents without diabetes agreed that people should get more information about diabetes and about diabetes prevention [26]. The review article by Pieper et al. [27] found that many studies showed that women had greater health-related information needs than men; this result was not confirmed by our study. However, women did feel subjectively better informed than men about diabetes in general and on specific topics.

A review by Biernatzki et al. [49] shows that the sources of information used by people with diabetes differ according to the type of information sought. Doctors were important sources of information about all forms of treatment, whereas the internet and printed information seem to play a more important role when it comes to other issues. In our study, adults with diabetes most frequently cited their general practitioner as their most frequent source of information followed by printed information, diabetologists and the internet. Nevertheless, it is impossible to rule out the possibility that a respondent’s general practitioner may have also been a diabetologist. Although the DAWN-2 study collected data on aspects that are relevant to the sources of information used by people with diabetes, the results have yet to be presented in detail [48]. Our study showed that respondents without diabetes informed themselves most frequently about the condition via printed media. Finally, adults without diabetes once again cited their general practitioner or other doctor as their most frequent source of information on general health issues.

Respondents with and without diabetes stated that their ability to judge the trustworthiness and clarity of the information published in the media about diabetes was limited. This problem was particularly pronounced among people with low levels of education. Although our study cannot really be used to measure health literacy, these results are in line with a 2014 representative survey of the health literacy of the German-speaking resident population aged 15 years or above. It found that 54.3% of respondents had limited levels of health literacy when measured using the German-language long version of the European Health Literacy Survey Questionnaire (HLS-EU-Q47); the study also identified a clear educational gradient [25].

### 4.2 Strengths and limitations

A strength of the study is that it is the first to implement a special procedure to collect data through interviewing large and population-representative samples of both the general population and adults with diabetes on some key issues including disease perception, information-seeking behaviour and information needs. By applying weighting factors, it is possible to directly compare the two groups. In addition, data was collected on further target group-relevant issues as well as on sociodemographic and psychosocial characteristics. Thus, information is available for the analysis of population subgroups, such as in terms of levels of knowledge about diabetes and information needs by gender and education. Our study also provides for the prospect of more in-depth investigations, such as into the relationship between psychosocial factors and disease burden among patients with diabetes or about the factors related to patient satisfaction.

However, our study also faces a limitation because telephone health surveys target the German-speaking population over the age of 18 years. This excludes adults without a sufficient knowledge of German. This method, therefore, does not allow for the representative collection of data from people with a recent family history of migration. Furthermore, the study also produced a relatively low response rate (17.9%). However, it is important to note that the response rate was calculated as a percentage of all households that are theoretically reachable by telephone in Germany [36]. Moreover, survey research has recently shown that a low response rate does not necessarily result in biased results (increased non-response bias) [51].

A further limitation of the study consists in the fact that it was only possible to categorise the participants (as with or without diabetes) using the information provided by the respondents themselves on the question of whether they had been diagnosed with diabetes by a doctor. However, it has been demonstrated that self-reported information about clearly defined diseases such as diabetes provides a very similar rate to that of actual physician diagnoses [20].

It was not possible to use established and validated instruments for all research questions, as some of these were not yet available. Therefore, certain questions had to be newly developed for the study. In addition, due to the limited length of the questionnaire, not all relevant questions could be taken into account or posed in more detail. For example, data could not be directly collected about the objective knowledge of diabetes in people with diabetes. The same applies to the self-efficacy, in other words, the belief that people have the capacity to perform certain actions such as practicing a healthy diet and physical activity.

### 4.3 Practical implications and outlook

Overall, the study shows a clear need for target group-specific information and communications on diabetes. Thus, it identified significant gaps in knowledge among the general population that could be addressed as part of an informational and communications-based strategy. Although knowledge is not necessarily indicative of health promoting behaviour or results in changes to behaviour, it is a prerequisite for both. In addition, the topics that people with or without diabetes wish for more information about can be directly incorporated into the planning of educational activities. Further in-depth analyses of the data from the present study could also provide information about the issues and sources of information that are relevant to specific target groups, such as people of certain age groups. The results indicate that it is not enough to provide information about diabetes merely on the internet, and that it is still needed in print. The involvement of doctors treating people with diabetes is also essential, as they constitute important contacts for diabetes-related issues as part of educational and communications strategies. Extended and in-depth analyses of the data that has been collected (Annex 1) are necessary to identify barriers and other factors that influence the use of disease prevention and care services, as these play an important role alongside knowledge.

Finally, the study also provides for the possibility for follow-up studies to be conducted as part of the National Diabetes Surveillance under the scientific coordination of the RKI. A repeat of the current survey, for example, could provide an important source of information for the Diabetes Surveillance in Germany and provide a basis with which to assess the success of the educational and communications-related measures being put in place by the BZgA.

## Key statements

- Around half of adults without diabetes and over 90% of adults with diabetes rate their knowledge of the condition as ‘good’ or ‘very good’.
- Almost one third of respondents without diabetes have already informed themselves about diabetes.
- Respondents without diabetes were particularly in need of information about ‘lifestyle changes, health promotion and disease prevention’.
- Respondents with diabetes were particularly in need of information about the ‘treatment and therapy’ of diabetes.
- About half of respondents found it difficult to judge the trustworthiness of the information about diabetes published in the media.

## Annex 1 Overview of the concepts and instruments employed in the study Disease knowledge and information needs - Diabetes mellitus (2017) Own diagram

**Table:**

Topic	Questionnaire used with participants without diabetes	Questionnaire used with participants with diabetes
**Knowledge about disease**		
Self-assessed level of knowledge about disease	1 item adapted from PIKS [1]	1 item adapted from PIKS [1]
Self-assessed level of knowledge about diabetes types	4 new items	-
Objective knowledge about disease	2 items adapted from Hoghton et al. [2]; four new items	-
Knowledge about diabetes due to occupation	1 new item	1 new item
**Disease perception**		
Type of disease and possibility of influencing it	1 item adapted from Adriaanse et al. [3], RPS-DD (‘personal control’ subscale, 4 items) [4]; 1 item adapted from IPQ-R for healthy individuals (timeline acute/chronic subscale) [5]	1 item adapted from Adriaanse et al. [3], IPQ-R (‘personal control’ subscale, 4 items) [6]; 1 item from IPQ-R for people with diabetes (timeline acute/chronic subscale) [6]
Stigmatisation	2 items adapted from Diabetes Ratgeber [7]; 1 new item based on DSAS-2 [8]	2 items adapted from Diabetes Ratgeber [7]; 1 new item based on DSAS-2 [8]
**Diabetes risk/risk of complications**		
Subjective risk	1 item adapted from Kim et al. [9], RPS-DD (‘optimistic bias’ subscale, 2 items) [4]	RPS-DM (‘optimistic bias’ subscale, 2 items) [10]
Objective diabetes risk	GDRS (18 Items) [11]	-
**Diabetes-related information**		
Perceived level of information	5 items from IND [12]	11 items from IND [12]
Subjective information needs	5 items from IND [12]	11 items from IND [12]
Information-seeking behaviour	7 items adapted from DAWN2 [13]	13 items adapted from DAWN2 [13]
Perceived trustworthiness and clarity of information	2 items from HLS-EU-Q16 [14]	2 items from HLS-EU-Q16 [14]
**Health-related behaviour and lifestyle**		
Physical activity, smoking, BMI	4 items from GDRS [11]	4 items from GDRS [11]
Use of health apps	1 item adapted from Ernsting et al. [15]	1 item adapted from Ernsting et al. [15]
**Health care services utilisation**		
Check-up 35	3 items adapted from DEGS1 [16], 2 new items	-
Disease management programmes (DMP), training	-	3 items adapted from DEGS1 [16], 8 items adapted from DAWN2 [17], 1 new item
Patient satisfaction	-	PACIC-DSF (9 Items) [17]
**Disease characteristics**		
Time of diagnosis, type of treatment, need for insulin, type of insulin delivery, type of blood glucose measurement, complications, comorbidities	-	Various items from DEGS1 [16] and GEDA 2014/2015-EHIS [18], partially adapted
**Diabetes self-management**		
Self-care behaviour	-	5 items adapted from SDSCA-6 [17]
Self-efficacy	-	4 items adapted from DCP [19]
**Psychosocial distress/burden in general**		
Diabetes distress	-	PAID-5 (5 Items) [17, 20]
Social support	-	DSDSP adapted (6 items) [17], one item adapted from DAWN2 [13]
Depressive symptoms	-	PHQ-2 (2 Items) [21]
Subjective health	5 items from MEHM [22]	4 items from MEHM [22]
Quality of life	1 item from GEDA 2014/2015-EHIS [18]	1 item from GEDA 2014/2015-EHIS [18]

**DAWN2**: Diabetes Attitudes Wishes and Needs 2; **DCP**: Diabetes Care Profile; **DEGS1**: German Health Interview and Examination Survey for Adults; **GDRS**: German Diabetes Risk Score; **DSAS-2**: Type 2 Diabetes Stigma Assessment Scale; **DSDSP**: DAWN Support for Diabetes Self-Management Profile; **GEDA 2014/2015-EHIS**: German Health Update 2014/2015-European Health Interview Survey; **HLS-EU-Q16**: European Health Literacy Survey Questionnaire (short form); **IND**: Information Needs in Diabetes Questionnaire; **IPQ-R**: Revised Illness Perception Questionnaire; **MEHM**: Minimum European Health Module; **PACIC-DSF**: Patient Assessment of Chronic Illness Care - DAWN Short Form; **PAID-5**: Problem Areas in Diabetes Scale 5; **PHQ-2**: Patient Health Questionnaire-2; **PIKS**: Perceived Kidney Knowledge Survey; **RPS-DD**: Risk Perception Survey Developing Diabetes; **RPS-DM**: Risk Perception Survey Diabetes Mellitus; **SDSCA-6**: Summary of Diabetes Self-Care Activities-6

## Annex 2 Questions analysed in the study Disease knowledge and information needs - Diabetes mellitus (2017) Own diagram

### Self-assessed level of knowledge about diabetes

How would you rate your understanding of diabetes?

**Possible answers:** very good, good, bad, non-existent

### Objective knowledge about diabetes

1. Diabetes is a disease of the blood cells.
2. Diabetes is a condition in which there is too much sugar in the blood.
3. Type 1 diabetes usually disappears with puberty.
4. Type 1 diabetes can be treated with tablets.
5. The symptoms of type 2 diabetes usually develop gradually.
6. In Germany, more people are affected by type 2 diabetes than type 1 diabetes.
  **Possible answers:** correct, false, don’t know

### Disease perception

**A) Respondents without diabetes**

1. How serious is diabetes in your view?
  **Possible answers:** not serious, moderately serious, serious, very serious, no opinion
2. I assume that people who have diabetes will have the condition for the rest of their life.
  **Possible answers:** completely disagree, disagree, neither agree nor disagree, agree, completely agree
3. Due to their illness, people with diabetes are often unable to meet their daily needs, for example, at work or in the family.
  **Possible answers:** completely disagree, disagree, neither agree nor disagree, agree, completely agree, don’t know
4. Most people who have diabetes have caused it themselves through an unhealthy lifestyle.
  **Possible answers:** completely disagree, disagree, neither agree nor disagree, agree, completely agree, don’t know
5. People with diabetes are often disadvantaged at work or privately.
  **Possible answers:** completely disagree, disagree, neither agree nor disagree, agree, completely agree, don’t know

**B) Respondents with diabetes**

1. How serious is diabetes in your view?
  **Possible answers:** not serious, moderately serious, serious, very serious, no opinion
2. I assume that I will have diabetes for the rest of my life.
  **Possible answers:** completely disagree, disagree, neither agree nor disagree, agree, completely agree
3. Other people often think that I will not be able to meet my daily needs, for example, at work or in the family because I have diabetes.
  **Possible answers:** completely disagree, disagree, neither agree nor disagree, agree, completely agree, don’t know
4. Other people think that I caused my diabetes due to my unhealthy lifestyle.
  **Possible answers:** completely disagree, disagree, neither agree nor disagree, agree, completely agree, don’t know
5. I am often disadvantaged at work or in my personal life because I have diabetes.
  **Possible answers:** completely disagree, disagree, neither agree nor disagree, agree, completely agree, don’t know

### Diabetes-related information

**(I) Perceived level of information**

How well informed are you about the following topics?

1. Causes of diabetes
2. Course of the disease
3. Treatment and therapy
4. Complications
5. Lifestyle changes, health promotion and disease prevention
6. Only respondents with diabetes: support services, helplines and sources of information
  **Possible answers:** very well, well, not well, not informed at all

**(II) Subjective information needs**

Would you currently like information on the following topics?

1. Causes of diabetes
2. Course of the disease
3. Treatment and therapy
4. Complications
5. Lifestyle changes, health promotion and disease prevention
6. Only respondents with diabetes: support services, helplines and sources of information
  **Possible answers:** yes, no

**(III) Information-seeking behaviour**

**A) Respondents without diabetes**

Which of the following sources of information have you used to find out about diabetes/health issues in general?

1. Your general practitioner or other doctors
2. A pharmacist
3. Printed information such as brochures, newspapers, magazines, books
4. Radio or television broadcasts
5. Websites about health and diabetes
6. Forums or social networks on the internet
7. Other sources of information than those just mentioned
  **Possible answers:** yes, no (Several answers possible)

**B) Respondents with diabetes**

Which of the following sources of information have you used to find out about diabetes?

1. A diabetologist
2. A diabetes advisor
3. Your general practitioner or other doctors
4. A pharmacist
5. Self-help groups where you meet other people with diabetes and/or their relatives
6. Natural health practitioners or doctors who practise alternative medicine, such as herbal medicine, acupuncture or homeopathy
7. People with diabetes in your social environment who advise or assist people with diabetes
8. Printed information such as brochures, newspapers, magazines, books
9. Information programs for people with diabetes such as those on the radio, television or DVD
10. Websites about health and diabetes
11. Forums or social networks on the internet
12. Diabetes hotlines or medical hotlines from your health insurance provider
13. Other sources of information than those just mentioned
  **Possible answers:** yes, no (Several answers possible)

**(IV) Self-assessed trustworthiness and clarity of information**

1. How easy do you think it is to judge whether the information about diabetes in the media is trustworthy?
  **Possible answers:** very easy, quite easy, quite difficult, very difficult
2. How easy do you think it is to understand the information about diabetes in the media?
  **Possible answers:** very easy, quite easy, quite difficult, very difficult

## Annex 3 Presentation of results of the study Disease knowledge and information needs - Diabetes mellitus (2017) according to gender

**Table 1:** Specific knowledge about diabetes among adults without diagnosed diabetes according to gender Source: Disease knowledge and information needs - Diabetes mellitus (2017)

Correct				False		Don’t know	
n		%	(95% CI)	%	(95% CI)	%	(95% CI)
**Diabetes is a disease of the blood cells**							
**Total**	2.323	14.5	(12.3-16.9)	31.3^*^	(28.7-34.0)	54.3	(51.3-57.2)
Women	1.311	13.5	(11.0-16.5)	33.9^*^	(30.5-37.5)	52.6	(48.7-56.5)
Men	1.012	15.5	(12.1-19.5)	28.4^*^	(24.7-32.6)	56.1	(51.5-60.6)
**Diabetes involves having too much sugar in the blood**							
**Total**	2.325	66.2^*^	(63.3-69.0)	11.8	(9.9-13.9)	22.0	(19.6-24.7)
Women	1.312	65.6^*^	(61.7-69.3)	11.9	(9.3-15.1)	22.5	(19.4-25.9)
Men	1.013	66.9^*^	(62.4-71.0)	11.6	(9.2-14.6)	21.5	(17.9-25.7)
**Type 1 diabetes disappears with puberty**							
**Total**	2.327	8.2	(6.7-10.0)	26.9^*^	(24.4-29.5)	64.9	(62.1-67.7)
Women	1.313	8.0	(6.1-10.3)	32.9^*^	(29.4-36.7)	59.1	(55.2-62.9)
Men	1.014	8.5	(6.2-11.4)	20.4^*^	(17.1-24.1)	71.2	(67.0-75.0)
**Type 1 diabetes can be treated with tablets**							
**Total**	2.326	36.3	(33.4-39.2)	17.2^*^	(15.0-19.6)	46.6	(43.6-49.6)
Women	1.312	37.2	(33.5-41.0)	22.5^*^	(19.3-26.1)	40.3	(36.5-44.1)
Men	1.014	35.2	(31.0-39.7)	11.4^*^	(8.9-14.6)	53.3	(48.7-57.8)
**Symptoms of type 2 diabetes usually develop slowly**							
**Total**	2.327	62.6^*^	(59.6-65.6)	1.4	(0.8-2.5)	35.9	(33.0-39.0)
Women	1.313	64.9^*^	(61.0-68.7)	1.4	(0.7-2.7)	33.7	(29.9-37.6)
Men	1.014	60.2^*^	(55.5-64.7)	1.5	(0.6-3.6)	38.4	(33.9-43.0)
**In Germany, more people are affected by type 2 diabetes than type 1 diabetes**							
**Total**	2.325	49.6^*^	(46.6-52.6)	2.5	(1.6-3.9)	47.9	(45.0-50.9)
Women	1.312	55.2^*^	(51.2-59.1)	1.6	(0.9-2.9)	43.2	(39.4-47.2)
Men	1.013	43.6^*^	(39.2-48.1)	3.4	(1.8-6.2)	53.0	(48.4-57.5)

* Indicates the correct answer

CI: Confidence interval

**Table 2:** Disease perception and stigmatisation among adults without diagnosed diabetes according to gender Source: Disease knowledge and information needs-Diabetes mellitus (2017)

Total			Women		Men	
%		(95% CI)	%	(95% CI)	%	(95% CI)
**Disease perception**						
**Diabetes is …**		(n=2.314)		(n=1.304)		(n=1.010)
Not serious	1.2	(0.8-1.9)	1.2	(0.6-2.2)	1.3	(0.7-2.4)
Moderately serious	21.3	(18.9-23.8)	20.1	(17.2-23.4)	22.5	(19.0-26.4)
Serious	48.1	(45.1-51.1)	51.2	(47.3-55.1)	44.7	(40.2-49.2)
Very serious	16.9	(14.7-19.2)	17.9	(15.2-20.9)	15.7	(12.5-19.6)
No opinion	12.6	(10.5-15.1)	9.6	(7.2-12.7)	15.8	(12.4-19.9)
**People have diabetes for the rest of their life**		(n=2.213)		(n=1.244)		(n=969)
Completely disagree	4.9	(3.6-6.6)	5.1	(3.4-7.5)	4.7	(2.9-7.3)
Disagree	23.3	(20.9-25.9)	24.1	(20.9-27.5)	22.5	(18.9-26.5)
Neither agree nor disagree	11.7	(9.9-13.9)	13.8	(11.0-17.2)	9.6	(7.3-12.4)
Agree	42.9	(39.9-46.0)	40.7	(36.8-44.7)	45.3	(40.7-50.0)
Completely agree	17.2	(14.9-19.7)	16.4	(13.6-19.6)	18.0	(14.5-22.2)
**Stigmatisation: People with diabetes …**						
**Are usually unable to meet their daily needs.**		(n=2.318)		(n=1.311)		(n=1.007)
Completely disagree	11.9	(10.2-13.9)	13.2	(10.8-16.1)	10.6	(8.3-13.4)
Disagree	41.6	(38.7-44.6)	42.4	(38.6-46.2)	40.8	(36.4-45.5)
Neither agree nor disagree	9.4	(7.9-11.2)	9.5	(7.5-12.0)	9.2	(7.0-12.1)
Agree	17.9	(15.7-20.4)	15.6	(12.9-18.7)	20.4	(16.9-24.5)
Completely agree	2.7	(1.8-4.0)	2.6	(1.5-4.7)	2.7	(1.5-4.9)
Don’t know	16.4	(14.2-18.9)	16.6	(13.7-20.0)	16.2	(13.0-19.9)
**Have caused their condition through an unhealthy lifestyle.**		(n=2.312)		(n=1.305)		(n=1.007)
Completely disagree	5.8	(4.7-7.3)	6.3	(4.6-8.6)	5.3	(3.9-7.2)
Disagree	33.4	(30.6-36.3)	34.1	(30.6-37.9)	32.6	(28.4-37.2)
Neither agree nor disagree	18.9	(16.7-21.4)	18.9	(16.0-22.2)	18.9	(15.6-22.7)
Agree	25.1	(22.7-27.8)	25.1	(21.9-28.6)	25.2	(21.4-29.3)
Completely agree	3.7	(2.7-5.1)	3.5	(2.1-5.7)	4.0	(2.7-6.1)
Don’t know	12.9	(11.0-15.2)	12.0	(9.5-15.0)	14.0	(11.0-17.6)
**Often face disadvantages.**		(n=2.315)		(n=1.311)		(n=1.004)
Completely disagree	9.6	(7.9-11.6)	9.3	(7.2-11.9)	9.9	(7.4-13.1)
Disagree	45.2	(42.3-48.2)	47.5	(43.6-51.4)	42.8	(38.4-47.4)
Neither agree nor disagree	8.5	(7.0-10.2)	7.9	(6.2-10.2)	9.0	(6.8-12.0)
Agree	12.9	(10.8-15.3)	10.7	(8.3-13.6)	15.2	(11.9-19.3)
Completely agree	1.1	(0.6-2.0)	1.4	(0.7-3.1)	0.8	(0.4-1.6)
Don’t know	22.7	(20.3-25.3)	23.2	(20.0-26.7)	22.2	(18.7-26.3)

CI: Confidence interval

**Table 3:** Perceived level of information among adults without diagnosed diabetes according to gender Source: Disease knowledge and information needs - Diabetes mellitus (2017)

Total			Women		Men	
%		(95% CI)	%	(95% CI)	%	(95% CI)
**Level of information**						
**Causes of diabetes**		(n=2.312)		(n=1.306)		(n=1.006)
Very well	4.5	(3.6-5.7)	6.2	(4.8-8.1)	2.7	(1.7-4.1)
Well	39.5	(36.7-42.4)	43.0	(39.2-46.9)	35.7	(31.6-40.2)
Not well	36.5	(33.7-39.5)	30.7	(27.3-34.4)	42.8	(38.3-47.4)
Not informed at all	19.4	(17.0-22.1)	20.0	(16.9-23.6)	18.8	(15.2-22.9)
**Course of disease**		(n=2.312)		(n=1.304)		(n=1.008)
Very well	4.2	(3.2-5.6)	5.2	(3.9-7.0)	3.2	(1.8-5.6)
Well	32.4	(29.7-35.1)	37.2	(33.5-41.0)	27.2	(23.6-31.2)
Not well	43.1	(40.1-46.1)	37.9	(34.2-41.8)	48.7	(44.1-53.3)
Not informed at all	20.3	(17.9-23.0)	19.7	(16.6-23.3)	20.9	(17.3-25.1)
**Treatment and therapy**		(n=2.312)		(n=1.304)		(n=1.008)
Very well	4.2	(3.3-5.4)	5.4	(4.0-7.1)	3.0	(1.9-4.7)
Well	37.0	(34.2-39.9)	42.7	(38.8-46.6)	31.0	(27.2-35.1)
Not well	39.7	(36.8-42.7)	35.1	(31.4-39.0)	44.6	(40.1-49.2)
Not informed at all	19.1	(16.7-21.7)	16.8	(14.1-20.0)	21.4	(17.7-25.8)
**Complications**		(n=2.307)		(n=1.301)		(n=1.006)
Very well	5.0	(3.9-6.3)	6.5	(4.9-8.6)	3.3	(2.1-5.1)
Well	31.0	(28.4-33.7)	35.1	(31.6-38.9)	26.5	(22.8-30.6)
Not well	40.7	(37.7-43.7)	38.4	(34.7-42.4)	43.0	(38.6-47.6)
Not informed at all	23.4	(20.8-26.3)	19.9	(16.8-23.4)	27.2	(23.0-31.8)
**Lifestyle changes, health promotion and disease prevention**		(n=2.301)		(n=1.295)		(n=1.006)
Very well	4.9	(3.9-6.2)	6.7	(5.1-8.7)	3.1	(2.0-5.0)
Well	44.0	(41.1-47.0)	45.9	(42.0-49.8)	42.1	(37.7-46.6)
Not well	33.6	(30.7-36.5)	30.1	(26.4-34.0)	37.3	(32.9-41.9)
Not informed at all	17.5	(15.2-20.0)	17.4	(14.5-20.8)	17.5	(14.1-21.5)

CI: Confidence interval

**Table 4:** Disease perception and stigmatisation in adults with diagnosed diabetes according to gender Source: Disease knowledge and information needs - Diabetes mellitus (2017)

Total			Women		Men	
%		(95% CI)	%	(95% CI)	%	(95% CI)
**Disease perception**						
**Diabetes is …**		(n=1.469)		(n=737)		(n=732)
Not serious	9.4	(7.8-11.2)	11.0	(8.6-13.9)	7.6	(5.8-10.0)
Moderately serious	27.1	(24.3-30.1)	26.6	(22.7-30.9)	27.7	(23.8-31.9)
Serious	45.8	(42.5-49.0)	43.6	(39.2-48.1)	48.1	(43.5-52.7)
Very serious	9.9	(8.2-12.0)	9.3	(7.2-12.1)	10.6	(7.9-14.0)
No opinion	7.8	(6.1-10.0)	9.5	(6.8-13.2)	6.0	(4.2-8.5)
**I will have diabetes for the rest of my life.^*^**		(n=1.386)		(n=671)		(n=715)
Completely disagree	1.4	(0.7-2.8)	1.4	(0.5-3.8)	1.5	(0.6-3.6)
Disagree	2.9	(1.7-4.9)	4.2	(2.1-8.3)	1.6	(0.9-2.8)
Neither agree nor disagree	4.7	(3.3-6.6)	4.2	(2.6-6.6)	5.1	(3.1-8.4)
Agree	45.1	(41.8-48.4)	44.6	(40.0-49.2)	45.5	(40.9-50.2)
Completely agree	46.0	(42.7-49.3)	45.6	(41.0-50.4)	46.3	(41.7-51.0)
**Stigmatisation**						
**Other people think that I cannot meet my daily needs because I have diabetes.^*^**		(n=1.382)		(n=668)		(n=714)
Completely disagree	33.5	(30.6-36.6)	37.7	(33.4-42.3)	29.4	(25.5-33.6)
Disagree	37.4	(34.3-40.6)	38.5	(34.0-43.2)	36.3	(32.1-40.8)
Neither agree nor disagree	4.0	(3.0-5.5)	2.9	(1.7-4.7)	5.2	(3.5-7.7)
Agree	11.4	(9.2-13.9)	7.0	(5.1-9.6)	15.7	(12.1-20.1)
Completely agree	3.5	(2.2-5.6)	4.4	(2.3-8.4)	2.7	(1.5-4.6)
Don’t know	10.1	(8.2-12.4)	9.5	(7.2-12.5)	10.7	(7.8-14.5)
**Other people think that I have caused my diabetes through my unhealthy lifestyle.^*^**		(n=1.377)		(n=673)		(n=704)
Completely disagree	26.5	(23.8-29.4)	29.5	(25.6-33.7)	23.5	(19.8-27.7)
Disagree	32.3	(29.3-35.5)	34.5	(30.2-39.2)	30.1	(26.2-34.4)
Neither agree nor disagree	7.0	(5.5-8.9)	5.9	(4.1-8.5)	8.1	(5.9-11.1)
Agree	17.3	(14.8-20.1)	12.6	(9.8-15.9)	22.0	(18.1-25.5)
Completely agree	5.0	(3.4-7.3)	4.5	(2.2-8.6)	5.6	(3.7-8.3)
Don’t know	11.9	(9.9-14.1)	13.0	(10.3-16.4)	10.7	(8.1-14.0)
**I often face disadvantages because I have diabetes.^*^**		(n=1.392)		(n=674)		(n=718)
Completely disagree	42.0	(38.8-45.3)	44.5	(39.9-49.3)	39.5	(35.1-44.0)
Disagree	46.7	(43.4-50.0)	46.5	(41.8-51.2)	46.9	(42.2-51.5)
Neither agree nor disagree	2.4	(1.6-3.7)	1.9	(1.1-3.3)	3.0	(1.6-5.4)
Agree	4.5	(3.4-6.0)	4.2	(2.7-6.4)	4.9	(3.3-7.2)
Completely agree	1.6	(0.8-3.1)	1.0	(0.4-2.6)	2.1	(0.8-5.3)
Don’t know	2.8	(1.8-4.3)	1.9	(1.1-3.5)	3.7	(2.1-6.5)

^*^ Indicates questions that were asked only to participants with diabetes in the last 12 months

CI: Confidence interval

**Table 5:** Perceived level of information among adults with diagnosed diabetes according to gender Source: Disease knowledge and information needs - Diabetes mellitus (2017)

Total			Women		Men	
%		(95% CI)	%	(95% CI)	%	(95% CI)
**Level of information**						
**Causes of diabetes**		(n=1.471)		(n=733)		(n=738)
Very well	17.1	(14.9-19.6)	15.8	(12.9-19.1)	18.6	(15.4-22.3)
Well	65.4	(62.3-68.4)	64.8	(60.5-68.9)	66.0	(61.5-70.3)
Not well	11.5	(9.6-13.7)	10.8	(8.5-13.7)	12.2	(9.3-15.9)
Not informed at all	6.0	(4.7-7.7)	8.6	(6.4-11.5)	3.1	(2.0-5.0)
**Course of disease**		(n=1.470)		(n=734)		(n=736)
Very well	16.1	(13.9-18.5)	14.2	(11.4-17.4)	18.1	(14.9-21.9)
Well	68.3	(65.3-71.2)	65.9	(61.5-70.0)	71.0	(66.7-75.0)
Not well	10.5	(8.7-12.7)	12.8	(10.1-16.1)	8.0	(5.8-11.0)
Not informed at all	5.1	(3.8-6.7)	7.2	(5.1-10.0)	2.8	(1.7-4.5)
**Treatment and therapy**		(n=1.469)		(n=734)		(n=735)
Very well	17.1	(14.9-19.6)	15.4	(12.5-18.8)	19.1	(15.8-22.8)
Well	67.6	(64.5-70.5)	67.6	(63.2-71.7)	67.5	(63.1-71.6)
Not well	10.3	(8.4-12.6)	10.7	(8.0-14.2)	9.8	(7.3-13.0)
Not informed at all	5.0	(3.8-6.6)	6.3	(4.4-8.9)	3.6	(2.2-5.8)
**Complications**		(n=1.467)		(n=736)		(n=731)
Very well	16.1	(13.9-18.6)	14.7	(11.9-18.1)	17.7	(14.4-21.4)
Well	63.2	(60.0-66.2)	60.8	(56.3-65.1)	65.8	(61.4-69.9)
Not well	14.4	(12.3-16.8)	16.6	(13.5-20.3)	12.0	(9.5-15.0)
Not informed at all	6.3	(5.0-7.9)	7.9	(5.8-10.5)	4.6	(3.1-6.7)
**Lifestyle changes, health promotion and disease prevention**		(n=1.459)		(n=730)		(n=729)
Very well	12.5	(10.6-14.8)	11.0	(8.5-14.0)	14.2	(11.3-17.7)
Well	59.7	(56.5-62.9)	59.1	(54.5-63.5)	60.5	(55.9-64.9)
Not well	16.9	(14.5-19.6)	15.9	(12.8-19.5)	18.0	(14.5-22.1)
Not informed at all	10.9	(9.0-13.1)	14.1	(11.0-17.9)	7.3	(5.4-9.8)
**Support services, helplines, information sources**		(n=1.451)		(n=730)		(n=721)
Very well	8.6	(7.0-10.5)	7.2	(5.3-9.6)	10.2	(7.7-13.3)
Well	48.5	(45.3-51.8)	47.7	(43.2-52.3)	49.5	(44.8-54.1)
Not well	26.5	(23.6-29.5)	25.9	(22.1-30.1)	27.1	(23.0-31.6)
Not informed at all	16.4	(14.2-18.9)	19.2	(16.1-22.8)	13.3	(10.3-16.9)

CI: Confidence interval

## Annex 4 Presentation of results of the study Disease knowledge and information needs - Diabetes mellitus (2017) according to educational status

**Table 1:** Specific knowledge about diabetes among adults without diagnosed diabetes according to educational status Source: Disease knowledge and information needs - Diabetes mellitus (2017)

Correct				False		Don’t know	
n		%	(95% CI)	%	(95% CI)	%	(95% CI)
**Diabetes is a disease of the blood cells**							
**Education**							
Low	337	19.0	(14.2-25.1)	14.9^*^	(10.8-20.2)	66.1	(59.5-72.1)
Middle	986	14.1	(11.3-17.5)	32.5^*^	(28.7-36.6)	53.3	(49.0-57.6)
High	996	9.8	(7.3-13.1)	47.7^*^	(43.0-52.5)	42.4	(37.8-47.2)
**Diabetes involves having too much sugar in the blood**							
**Education**							
Low	338	62.9^*^	(56.3-69.0)	7.1	(4.2-11.8)	30.0	(24.3-36.4)
Middle	988	67.2^*^	(63.1-71.2)	12.7	(10.0-16.0)	20.1	(16.9-23.6)
High	995	68.3^*^	(63.8-72.5)	15.7	(12.4-19.8)	15.9	(13.1-19.2)
**Type 1 diabetes disappears with puberty**							
**Education**							
Low	338	7.2	(4.5-11.2)	17.7^*^	(13.3-23.1)	75.2	(69.2-80.3)
Middle	988	9.8	(7.7-12.5)	26.3^*^	(22.8-30.3)	63.8	(59.6-67.8)
High	997	6.9	(4.5-10.4)	38.0^*^	(33.5-42.7)	55.2	(50.4-59.9)
**Type 1 diabetes can be treated with tablets**							
**Education**							
Low	337	39.7	(33.6-46.1)	10.0^*^	(6.4-15.1)	50.3	(43.9-56.8)
Middle	988	39.1	(35.0-43.4)	16.6^*^	(13.5-20.3)	44.3	(40.0-48.7)
High	997	28.0	(24.2-32.0)	26.0^*^	(21.9-30.5)	46.1	(41.3-50.9)
**Symptoms of type 2 diabetes usually develop slowly**							
**Education**							
Low	338	50.6^*^	(44.1-57.0)	1.4	(0.3-5.8)	48.1	(41.6-54.5)
Middle	988	66.5^*^	(62.2-70.5)	1.1	(0.6-2.2)	32.4	(28.4-36.7)
High	997	70.7^*^	(66.1-74.9)	1.9	(0.9-4.3)	27.4	(23.3-31.8)
**In Germany, more people are affected by type 2 diabetes than type 1 diabetes**							
**Education**							
Low	338	37.1^*^	(31.3-43.4)	2.2	(0.7-6.2)	60.7	(54.3-66.7)
Middle	987	52.3^*^	(47.9-56.7)	3.0	(1.6-5.6)	44.7	(40.4-49.1)
High	996	59.5^*^	(54.8-64.1)	1.8	(1.0-3.4)	38.6	(34.1-43.3)

* Indicates the correct answer

CI: Confidence interval

**Table 2:** Disease perception and stigmatisation among adults without diagnosed diabetes according to educational status Source: Disease knowledge and information needs - Diabetes mellitus (2017)

Low			Middle		High	
%		(95% CI)	%	(95% CI)	%	(95% CI)
**Disease perception**						
**Diabetes is …**		(n=334)		(n=985)		(n=991)
Not serious	1.1	(0.4-3.0)	1.3	(0.7-2.3)	1.3	(0.5-3.0)
Moderately serious	14.0	(10.4-18.5)	24.4	(20.8-28.3)	24.7	(20.5-29.5)
Serious	47.8	(41.4-54.3)	48.3	(44.0-52.7)	47.6	(42.9-52.3)
Very serious	19.5	(14.8-25.3)	14.4	(11.7-17.7)	17.7	(14.5-21.5)
No opinion	17.6	(12.7-24.0)	11.6	(9.0-14.8)	8.6	(6.0-12.4)
**People have diabetes for the rest of their lives.^*^**		(n=307)		(n=950)		(n=952)
Completely disagree	5.4	(2.8-10.4)	4.1	(2.6-6.3)	5.6	(3.8-8.2)
Disagree	23.7	(18.7-29.7)	22.2	(18.9-25.9)	24.5	(20.4-29.2)
Neither agree nor disagree	9.8	(6.4-14.7)	12.9	(10.0-16.4)	12.1	(9.4-15.4)
Agree	41.5	(35.0-48.3)	45.0	(40.6-49.5)	40.9	(36.3-45.7)
Completely agree	19.5	(14.7-25.5)	15.8	(12.8-19.3)	16.9	(13.2-21.4)
**Stigmatisation: People with diabetes …**						
**Are usually unable to meet their daily needs.^*^**		(n=337)		(n=988)		(n=989)
Completely disagree	7.0	(4.6-10.5)	14.2	(11.4-17.5)	14.2	(11.2-17.8)
Disagree	31.0	(25.3-37.3)	44.9	(40.6-49.3)	48.9	(44.1-53.6)
Neither agree nor disagree	10.0	(6.8-14.6)	7.7	(5.8-10.0)	11.5	(8.9-14.8)
Agree	23.2	(18.3-28.9)	16.9	(13.8-20.5)	13.3	(10.2-17.0)
Completely agree	5.1	(2.7-9.4)	1.7	(1.0-3.1)	1.4	(0.7-2.8)
Don’t know	23.7	(18.5-29.9)	14.6	(11.9-17.9)	10.8	(8.2-14.0)
**Have caused their diabetes through an unhealthy lifestyle.^*^**		(n=337)		(n=985)		(n=986)
Completely disagree	3.7	(2.0-6.8)	6.6	(4.8-9.0)	6.6	(4.6-9.5)
Disagree	30.3	(24.7-36.5)	34.1	(30.1-38.3)	36.1	(31.4-41.1)
Neither agree nor disagree	14.1	(10.2-19.3)	22.4	(18.7-26.6)	19.0	(16.1-22.4)
Agree	26.7	(21.4-32.6)	24.7	(21.1-28.6)	24.3	(20.6-28.4)
Completely agree	6.2	(3.7-10.3)	2.4	(1.4-4.1)	3.0	(1.8-4.9)
Don’t know	19.0	(14.2-24.9)	9.8	(7.7-12.3)	10.9	(8.1-14.6)
**Often face disadvantages.^*^**		(n=334)		(n=987)		(n=990)
Completely disagree	8.3	(4.9-13.5)	10.1	(7.8-13.0)	10.5	(8.1-13.5)
Disagree	37.6	(31.6-43.9)	49.4	(45.0-53.7)	47.6	(42.8-52.3)
Neither agree nor disagree	8.0	(5.0-12.7)	8.2	(6.3-10.7)	9.0	(6.9-11.8)
Agree	13.9	(9.8-19.2)	13.0	(9.8-19.2)	11.6	(8.9-15.1)
Completely agree	1.7	(0.7-4.1)	0.8	(0.4-1.7)	1.0	(0.3-3.9)
Don’t know	30.6	(25.0-36.8)	18.6	(15.5-22.1)	20.3	(16.7-24.4)

*Indicates questions that were asked only to participants with diabetes in the last 12 months

CI: Confidence interval

**Table 3:** Self-assessed level of knowledge about diabetes and perceived level of information among adults without diagnosed diabetes according to educational status Source: Disease knowledge and information needs - Diabetes mellitus (2017)

Low			Middle		High	
%		(95% CI)	%	(95% CI)	%	(95% CI)
**Self-assessed level of knowledge about diabetes**		(n=331)		(n=983)		(n=995)
Very good	5.7	(3.4-9.5)	9.2	(7.3-11.5)	17.0	(13.6-21.1)
Good	43.0	(36.8-49.4)	49.6	(45.2-54.0)	45.6	(41.0-50.3)
Bad	31.1	(25.4-37.4)	33.5	(29.4-37.9)	30.4	(26.1-35.1)
Non-existent	20.2	(15.0-26.6)	7.7	(5.4-10.9)	7.0	(4.6-10.5)
**Perceived level of information about …**						
**Causes of diabetes**		(n=331)		(n=986)		(n=991)
Very well	2.3	(1.1-4.7)	4.1	(2.9-5.8)	7.8	(5.7-10.5)
Well	27.6	(22.2-33.8)	44.0	(39.7-48.3)	45.8	(41.2-50.6)
Not well	36.8	(30.8-43.3)	35.7	(31.6-40.0)	37.5	(32.9-42.3)
Not informed at all	33.3	(27.4-39.8)	16.3	(13.2-19.9)	8.9	(6.6-12.0)
**Course of disease**		(n=335)		(n=984)		(n=989)
Very well	3.5	(1.6-7.5)	3.5	(2.3-5.1)	6.3	(4.5-8.9)
Well	29.4	(24.0-35.5)	31.5	(27.9-35.4)	37.0	(32.5-41.7)
Not well	38.3	(32.2-44.8)	45.2	(40.9-49.7)	45.1	(40.4-49.9)
Not informed at all	28.8	(23.2-35.1)	19.8	(16.3-23.8)	11.6	(9.0-14.8)
**Treatment and therapy**		(n=333)		(n=984)		(n=991)
Very well	2.6	(1.2-5.4)	3.6	(2.5-5.2)	7.1	(5.1-9.8)
Well	30.7	(25.1-37.0)	39.2	(35.1-43.4)	40.7	(36.2-45.3)
Not well	38.6	(32.5-45.1)	39.4	(35.1-43.9)	41.4	(36.8-46.3)
Not informed at all	28.1	(22.6-34.3)	17.8	(14.7-21.4)	10.8	(7.8-14.6)
**Complications**		(n=331)		(n=985)		(n=988)
Very well	3.6	(1.8-7.3)	4.1	(2.8-5.8)	7.9	(5.8-10.6)
Well	26.9	(21.6-32.9)	29.8	(26.2-33.7)	37.2	(32.7-41.9)
Not well	36.4	(30.5-42.8)	42.7	(38.4-47.1)	42.4	(37.7-47.2)
Not informed at all	33.0	(27.1-39.6)	23.5	(19.8-27.6)	12.5	(9.7-15.9)
**Lifestyle changes, health promotion and disease prevention**		(n=329)		(n=981)		(n=987)
Very well	2.3	(0.8-6.0)	4.8	(3.4-6.7)	8.3	(6.2-10.9)
Well	34.0	(28.2-40.4)	45.5	(41.2-49.9)	52.8	(48.0-57.6)
Not well	37.2	(31.0-43.8)	32.6	(28.6-36.9)	31.0	(26.5-35.9)
Not informed at all	26.5	(21.2-32.6)	17.1	(13.8-20.9)	7.9	(5.8-10.6)

CI: Confidence interval

**Table 4:** Subjective information needs on diabetes-specific topics, trustworthiness and clarity of information among adults without diagnosed diabetes according to educational status Source: Disease knowledge and information needs - Diabetes mellitus (2017)

Low			Middle		High	
%		(95% CI)	%	(95% CI)	%	(95% CI)
**Subjective information needs**						
**Causes of diabetes**		(n=338)		(n=986)		(n=994)
Yes	41.4	(35.2-47.9)	52.9	(48.5-57.2)	42.0	(37.3-46.8)
**Course of disease**		(n=338)		(n=986)		(n=992)
Yes	40.7	(34.6-47.2)	49.5	(45.2-53.9)	36.5	(31.8-41.5)
**Treatment and therapy**		(n=337)		(n=986)		(n=994)
Yes	41.1	(34.9-47.5)	48.4	(44.1-52.8)	39.5	(34.8-44.4)
**Complications**		(n=337)		(n=986)		(n=994)
Yes	43.3	(37.0-49.7)	52.5	(48.2-56.8)	41.9	(37.2-46.8)
**Lifestyle changes, health promotion and disease prevention**		(n=336)		(n=986)		(n=994)
Yes	50.1	(43.7-56.6)	57.3	(53.0-61.5)	52.4	(47.7-57.1)
**Judge the trustworthiness of …**						
**Information about diabetes^*^**		(n=67)		(n=338)		(n=413)
Very easy	7.2	(3.2-15.3)	8.8	(5.3-14.3)	6.9	(4.0-11.5)
Quite easy	27.5	(17.7-40.1)	36.9	(30.4-43.8)	43.8	(36.7-51.2)
Quite difficult	58.0	(44.0-70.9)	47.3	(40.2-54.5)	44.6	(37.3-52.3)
Very difficult	7.3	(2.2-21.4)	7.1	(4.5-11.0)	4.7	(2.7-8.0)
**Judge the clarity of …**						
**Information about diabetes^*^**		(n=69)		(n=337)		(n=417)
Very easy	11.4	(4.6-25.5)	17.6	(12.5-24.3)	15.9	(11.6-21.5)
Quite easy	58.3	(43.7-71.6)	48.7	(41.7-55.8)	56.7	(49.2-64.0)
Quite difficult	25.7	(15.1-40.2)	30.2	(24.1-37.0)	25.1	(19.0-32.4)
Very difficult	4.6	(1.1-16.7)	3.5	(1.2-9.7)	2.3	(0.4-12.4)

* Indicates questions that were asked only to participants who have actively sought information about diabetes previously

CI: Confidence interval

**Table 5:** Disease perception and stigmatisation among adults with diagnosed diabetes according to educational status Source: Disease knowledge and information needs - Diabetes mellitus (2017)

Low			Middle		High	
%		(95% CI)	%	(95% CI)	%	(95% CI)
**Disease perception**						
**Diabetes is …**		(n=412)		(n=632)		(n=423)
Not serious	11.5	(8.8-14.9)	8.2	(6.2-10.8)	5.8	(3.8-8.6)
Moderately serious	26.8	(22.2-31.9)	25.2	(21.5-29.4)	33.5	(28.0-39.6)
Serious	43.1	(37.7-48.6)	48.8	(44.2-53.4)	46.0	(40.0-52.2)
Very serious	7.2	(4.9-10.5)	12.9	(10.1-16.4)	9.9	(6.2-15.4)
No opinion	11.5	(8.2-15.8)	4.8	(3.3-7.0)	4.7	(2.9-7.7)
**I will have diabetes for the rest of my life.^*^**		(n=390)		(n=591)		(n=403)
Completely disagree	2.2	(0.9-5.2)	0.8	(0.3-2.4)	0.5	(0.1-3.3)
Disagree	2.9	(1.0-8.0)	2.7	(1.7-4.4)	3.2	(1.3-7.4)
Neither agree nor disagree	5.0	(2.8-8.7)	3.5	(2.2-5.7)	7.0	(3.6-13.2)
Agree	49.2	(43.6-54.9)	43.1	(38.6-47.6)	36.5	(31.0-42.4)
Completely agree	40.6	(35.2-46.3)	49.9	(45.3-54.5)	52.8	(46.5-59.1)
**Stigmatisation**						
**Other people think that I cannot meet my daily needs because I have diabetes^*^**		(n=391)		(n=589)		(n=400)
Completely disagree	28.6	(24.0-33.7)	35.0	(30.8-39.5)	46.4	(40.0-52.9)
Disagree	39.9	(34.6-45.5)	36.6	(32.4-41.0)	31.5	(26.3-37.1)
Neither agree nor disagree	4.8	(2.9-7.7)	2.7	(1.7-4.3)	5.5	(3.2-9.3)
Agree	11.7	(8.3-16.3)	12.0	(9.0-15.8)	8.4	(5.6-12.5)
Completely agree	3.9	(1.8-8.4)	4.0	(2.4-6.5)	0.6	(0.2-1.7)
Don’t know	11.0	(7.8-15.4)	9.7	(7.2-13.0)	7.6	(5.1-11.2)
**Other people think that I have caused my diabetes through my unhealthy lifestyle.^*^**		(n=389)		(n=588)		(n=398)
Completely disagree	22.8	(18.5-27.6)	28.1	(24.2-32.4)	35.0	(29.1-41.4)
Disagree	31.8	(26.9-37.2)	33.9	(29.6-38.3)	29.5	(24.3-35.2)
Neither agree nor disagree	7.1	(4.7-10.7)	5.6	(3.8-8.2)	10.8	(7.4-15.4)
Agree	18.6	(14.4-23.6)	17.8	(14.4-21.7)	11.2	(8.2-15.3)
Completely agree	5.7	(3.1-10.4)	4.6	(2.9-7.3)	3.5	(1.8-6.7)
Don’t know	14.0	(10.6-18.2)	10.0	(7.7-12.9)	10.0	(7.2-13.7)
**I often face disadvantages because I have diabetes.^*^**		(n=392)		(n=594)		(n=404)
Completely disagree	39.1	(33.8-44.7)	43.5	(39.1-48.1)	47.7	(41.4-54.1)
Disagree	51.6	(46.0-57.2)	42.2	(37.8-46.8)	42.8	(36.8-49.0)
Neither agree nor disagree	1.9	(0.8-4.5)	3.5	(2.1-5.7)	1.4	(0.6-3.0)
Agree	2.6	(1.4-4.7)	6.4	(4.3-9.3)	5.5	(3.0-9.8)
Completely agree	1.8	(0.6-5.6)	1.6	(0.7-3.7)	0.6	(0.1-2.3)
Don’t know	3.1	(1.6-5.7)	2.8	(1.4-5.5)	2.1	(0.9-4.7)

* Indicates questions that were asked only to participants with diabetes in the last 12 months

CI: Confidence interval

**Table 6:** Perceived level of information among adults with diagnosed diabetes according to educational status Source: Disease knowledge and information needs – Diabetes mellitus (2017)

Low			Middle		High	
%		(95% CI)	%	(95% CI)	%	(95% CI)
**Level of information**						
**Causes of diabetes**		(n=412)		(n=630)		(n=427)
Very well	11.6	(8.6-15.5)	20.2	(16.8-24.1)	26.8	(21.7-32.6)
Well	66.5	(61.3-71.4)	64.7	(60.2-69.0)	63.3	(57.0-69.3)
Not well	13.8	(10.6-17.8)	9.7	(7.3-12.8)	9.0	(5.3-14.8)
Not informed at all	8.1	(5.8-11.2)	5.4	(3.6-8.0)	0.9	(0.3-2.8)
**Course of disease**		(n=410)		(n=632)		(n=426)
Very well	11.1	(8.1-15.1)	19.2	(15.9-23.0)	23.3	(18.4-29.1)
Well	72.3	(67.2-76.8)	65.1	(60.6-69.4)	64.7	(58.5-70.4)
Not well	10.0	(7.3-13.7)	11.1	(8.4-14.5)	10.4	(7.1-14.9)
Not informed at all	6.5	(4.5-9.4)	4.6	(2.9-7.5)	1.6	(0.7-3.7)
**Treatment and therapy**		(n=410)		(n=631)		(n=426)
Very well	12.9	(9.6-17.0)	18.8	(15.5-22.5)	26.8	(21.5-32.8)
Well	71.3	(66.1-76.0)	63.7	(59.1-68.0)	66.6	(60.4-72.3)
Not well	9.1	(6.3-12.9)	13.0	(10.0-16.8)	6.0	(3.7-9.8)
Not informed at all	6.8	(4.6-9.8)	4.5	(2.9-6.9)	0.6	(0.2-1.6)
**Complications**		(n=408)		(n=630)		(n=427)
Very well	11.0	(8.1-15.0)	19.2	(15.8-23.1)	24.0	(19.0-29.9)
Well	64.8	(59.6-69.7)	61.6	(57.0-66.0)	62.0	(55.8-67.8)
Not well	15.8	(12.4-19.9)	13.9	(10.9-17.5)	11.5	(8.0-16.2)
Not informed at all	8.4	(6.0-11.5)	5.3	(3.7-7.6)	2.5	(1.3-4.5)
**Lifestyle changes, health promotion and disease prevention**		(n=402)		(n=631)		(n=424)
Very well	9.7	(6.8-13.6)	13.8	(10.9-17.2)	18.3	(14.3-23.1)
Well	57.0	(51.4-62.4)	60.7	(56.2-65.1)	65.8	(59.4-71.7)
Not well	18.5	(14.5-23.2)	16.2	(13.0-19.9)	13.5	(8.8-20.1)
Not informed at all	14.8	(11.3-19.2)	9.3	(7.1-12.2)	2.5	(1.3-4.8)
**Support services, helplines, information sources**		(n=402)		(n=625)		(n=422)
Very well	6.7	(4.4-10.1)	8.4	(6.4-11.1)	15.4	(11.1-21.0)
Well	46.1	(40.6-51.7)	50.0	(45.4-54.5)	52.4	(46.2-58.6)
Not well	27.5	(22.9-32.7)	26.0	(22.1-30.4)	24.1	(18.8-30.3)
Not informed at all	19.7	(15.8-24.2)	15.6	(12.6-19.1)	8.1	(5.7-11.4)

CI: Confidence interval

**Table 7:** Subjective information needs on diabetes-specific topics, trustworthiness and clarity of information among adults with diagnosed diabetes according to educational status Source: Disease knowledge and information needs – Diabetes mellitus (2017)

Low			Middle		High	
%		(95% CI)	%	(95% CI)	%	(95% CI)
**Subjective information needs**						
**Causes of diabetes**		(n=413)		(n=633)		(n=427)
Yes	37.0	(31.9-42.3)	35.1	(30.9-39.6)	23.7	(18.5-29.7)
**Course of disease**		(n=413)		(n=634)		(n=427)
Yes	38.9	(33.9-44.2)	41.0	(36.6-45.6)	30.0	(24.5-36.2)
**Treatment and therapy**		(n=413)		(n=633)		(n=427)
Yes	46.6	(41.2-52.1)	54.3	(49.8-58.8)	39.3	(33.5-45.5)
**Complications**		(n=413)		(n=632)		(n=427)
Yes	42.7	(37.4-48.1)	49.3	(44.7-53.9)	35.8	(30.2-41.9)
**Lifestyle changes, health promotion and disease prevention**		(n=411)		(n=629)		(n=425)
Yes	40.5	(35.3-46.0)	43.6	(39.1-48.2)	33.7	(28.0-39.8)
**Support services, helplines, information sources**		(n=409)		(n=631)		(n=425)
Yes	42.8	(37.5-48.3)	48.5	(43.9-53.0)	37.6	(31.8-43.9)
**Judge the trustworthiness of …**						
**Information about diabetes**		(n=379)		(n=585)		(n=399)
Very easy	8.9	(6.0-13.0)	12.3	(9.1-16.3)	7.8	(5.3-11.4)
Quite easy	40.0	(34.5-45.8)	39.1	(34.7-43.7)	46.7	(40.5-53.0)
Quite difficult	38.8	(33.3-44.5)	41.2	(36.5-46.0)	38.1	(32.0-44.6)
Very difficult	12.3	(9.2-16.2)	7.4	(5.3-10.3)	7.4	(4.0-13.3)
**Judge the clarity of …**						
**Information about diabetes**		(n=383)		(n=603)		(n=406)
Very easy	12.7	(9.0-17.6)	12.8	(10.0-16.3)	17.7	(13.6-22.7)
Quite easy	47.0	(41.3-52.7)	48.3	(43.7-53.0)	63.6	(57.5-69.3)
Quite difficult	33.4	(28.4-38.8)	34.6	(30.2-39.4)	16.0	(12.0-21.1)
Very difficult	6.9	(4.5-10.5)	4.2	(2.7-6.4)	2.7	(1.3-5.4)

CI: Confidence interval
